# A Novel Wideband Transition from LTCC Laminated Waveguide to Air-Filled Rectangular Waveguide for W-band Applications

**DOI:** 10.3390/mi14010052

**Published:** 2022-12-25

**Authors:** Bin Yuan, Qing Du, Chengxiang Hao, Yan Zhao, Zhongjun Yu

**Affiliations:** 1 Aerospace Information Research Institute, Chinese Academy of Sciences, Beijing 100190, China; 2 School of Electronic, Electrical and Communication Engineering, University of Chinese Academy of Sciences, Beijing 100190, China; 3 School of Information and Communication Engineering, Communication University of China, Beijing 100024, China

**Keywords:** air-filled rectangular waveguide, laminated waveguide, low-temperature co-fired ceramic (LTCC), transition, W-band, wideband

## Abstract

In this paper, a novel wideband transition from a laminated waveguide (LWG) to an air-filled rectangular waveguide (RWG) is proposed for millimeter-wave integration solutions based on multilayer low-temperature co-fired ceramic (LTCC) technology. The integrated transition cavity is divided into several resonators by introducing five grounded via holes. Due to the magnetic wall existing in the symmetry plane, the equivalent circuit of the proposed transition can be simplified as a three-pole filter model to explain the working mechanism with wideband performance. A W-band integrated LWG-to-RWG transition is designed as an example using LTCC technology. Two back-to-back prototypes with different lengths are fabricated and measured. A measured 25.7% bandwidth from 76 GHz to 101 GHz can be achieved for return loss better than 14 dB. The average insertion loss of a single transition is about 0.5 dB. The compact structure and wideband performance give it potential in high-density millimeter-wave and terahertz packaging.

## 1. Introduction

With the gradual depletion of spectrum resources, the high-frequency bands of millimeter-wave (MMW) and even the terahertz (THz) band become increasing attractive in large-bandwidth wireless systems such as high-resolution radars and high-speed wireless communications [1]. The precision processing technology of print circuits is necessary to achieve advanced MMW/THz integrated modules and systems. An integrated solution based on a thin-film circuit process is a good choice for the THz band due to its low-loss and stable performance [2], but the single-layer scheme limits the design flexibility and circuit density. Low-temperature co-fired ceramic (LTCC) technology can highly integrate multiple devices in multilayer dielectric substrate, which meets the design requirements of high precision and miniaturization of MMW/THz electronic products [3]. Compact laminated waveguide (LWG) based on LTCC technology has been widely used to solve the problems of radiation loss of microstrip transmission lines in the high MMW band, which is suitable for MMW/THz integration and packaging. However, the signal interface of the MMW/THz packaging module is generally formed with an air-filled rectangular waveguide (RWG) due to the superior features of excellent durability and low insertion loss [4]. Therefore, the integrated transition between LWG and air-filled RWG is crucial for MMW/THz LTCC packaging modules.

In the past decades, various approaches have been presented to achieve the transition from a substrate-integrated waveguide to an air-filled RWG based on traditional printed circuit board (PCB) technology [5,6,7,8,9]. However, the permittivity of LTCC dielectric substrate is high. It is quite a challenge to obtain wideband performance with the transition from LWG to air-filled RWG due to the dimension mismatch and huge impedance difference between them. Several earlier works of LWG-to-RWG transitions based on LTCC technology have been reported [10,11,12,13]. The integrated LTCC prototype of LWG-to-RWG transition is first presented in [10] and gives an effective bandwidth of over 8% in Ka-band. To miniaturize the transition structure, a compact configuration is designed to take advantage of the limited volume in substrate near the air-filled RWG aperture [11]. In [12], an extra air cavity is introduced in the LTCC laminated substrate to improve impedance matching, expanding the simulated bandwidth to 10.8% with a compromise of fabrication complexity.

In this paper, a novel LWG-to-RWG transition with large bandwidth and compact size is proposed for W-band LTCC applications. To obtain wideband performance, five grounded via holes are strategically arranged in the transition cavity to generate a simplified three-pole filter model, which produces three reflection zeros in the passband. The integrated LTCC transition is fabricated and measured. Measured results are in good agreement with simulated ones.

## 2. Transition Design and Analysis

### 2.1. Configuration and Working Mechanism

**Figure 1:**
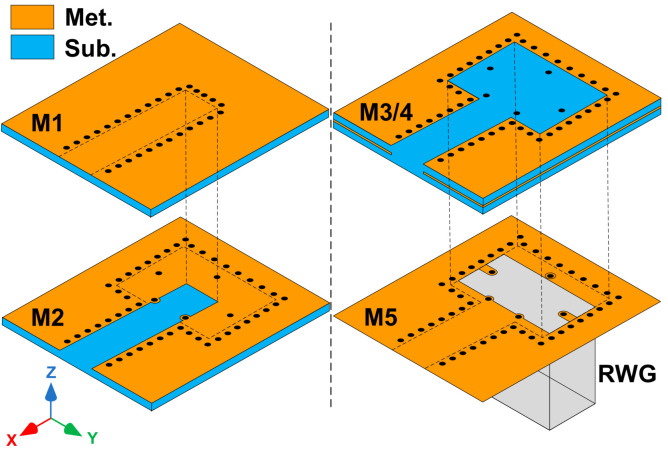
Exploded view of proposed transition with four layers of LTCC dielectric substrate.

Figure 1 gives the multilayer configuration of the proposed LTCC transition from the LWG to the air-filled RWG. The LTCC laminated waveguide consists of four dielectric layers and five metal layers and is directly mounted on the vertical RWG port. The aperture size of the bottom metal layer M5 is the same as the cross-section of a standard air-filled RWG. An enclosed three-layer (M2–M5) transition cavity above the RWG port is surrounded by a metalized via-hole fence to prevent energy leakage. The whole transition structure is designed to occupy as minimal a space as possible around the RWG port.

**Figure 2:**
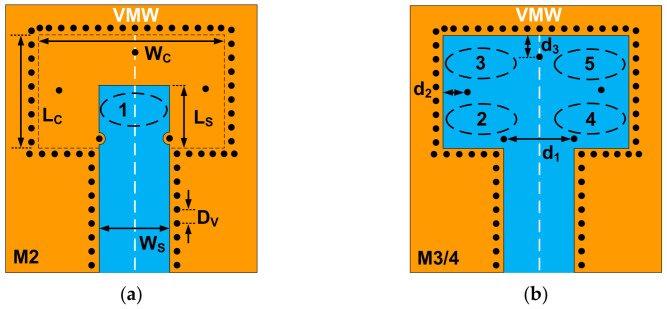
Top view of the proposed transition: (**a**) top metal layer M2; (**b**) middle metal layer M3 and M4.

**Figure 3:**
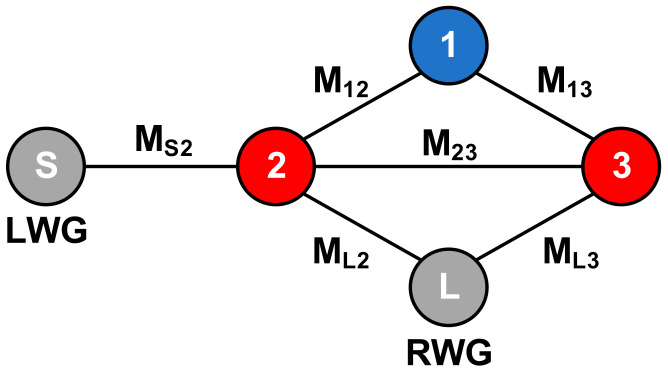
Equivalent filter model of the proposed transition.

As shown in Figure 2a, the LWG in the first layer is inserted above the transition cavity. The insertion length LS is about half the waveguide wavelength to create an equivalent resonator, marked as ‘1’. It can be seen from Figure 2b that five grounded via holes divide the three-layer transition cavity into four half-wavelength resonators marked as ‘2’, ‘3’, ‘4’, and ‘5’, respectively. The region and position of each equivalent resonator are denoted with dotted circles in Figure 2. Obviously, the whole transition is a symmetric structure, and there is a virtual magnetic wall (VMW) at the symmetry plane. Therefore, the resonators on both sides of the magnetic wall are isolated from each other, and no coupling power crosses the magnetic wall, which simplifies the equivalent filter model of the proposed transition. As illustrated in Figure 3, a three-pole filter model with an LWG source and an RWG load is created by three Resonators 1, 2, and 3, which can be used to predict the performance of the transition. The input LWG is coupled with Resonator 2 by a coefficient M*_S_*_2_. The coupling coefficients M_12_, M_13_, M_23_ represent the mutual coupling between the three resonators. Resonators 2 and 3 are coupled with the output air-filled RWG by coupling coefficients M*_L_*_2_ and M*_L_*_3_, respectively. According to the proposed filter topology and cross-coupled theory, there will be three reflection zeros in the passband, and two prescribed transmission zeros can be generated with an appropriate coupling matrix.

### 2.2. Design and Simulation

In order to validate the feasibility of the proposed transition scheme and the equivalent model, a W-band design is investigated. The height of the single-layer LTCC substrate is taken as 0.096 mm, and the thickness of the gold metal layer is 0.008 mm. Ferro A6M with permittivity of 5.9 and loss tangent of 0.002 is chosen as the dielectric material of the LTCC substrate. The standard rectangular waveguide WR10 of 2.54 mm × 1.27 mm is used in this design. Full-wave electromagnetic simulation is executed with the physical structure shown in Figure 1. The dimensions of the transition cavity *L_C_* and *W_C_* are crucial to the eigen frequency of Resonators 2 and 3, while the insertion length LS influences the eigen frequency of Resonator 1. The distance *d*_1_ between two via holes on the entrance of the transition cavity determines the coupling from the LWG port to Resonator 2. The distance *d*_2_ is a key parameter for the coupling between Resonator 2 and Resonator 3. In addition, a via hole with distance *d*_3_ from the cavity edge is arranged on the VMW plane to adjust the impedance matching.

**Figure 4:**
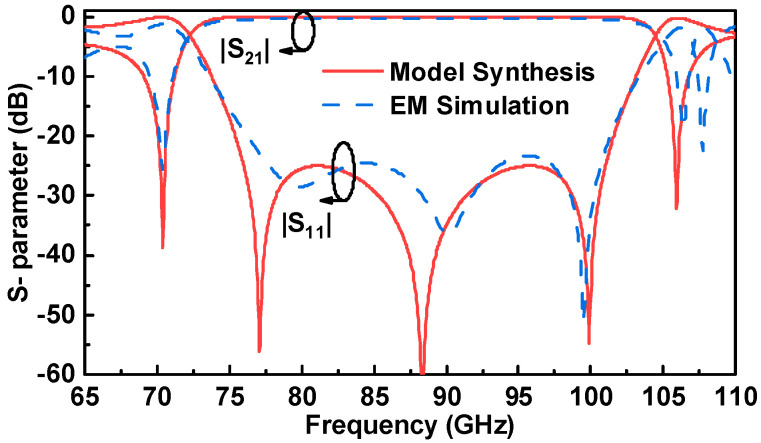
S-parameters comparison between classical filter synthesis and full-wave electromagnetic simulation of the proposed single transition.

**Figure 5:**
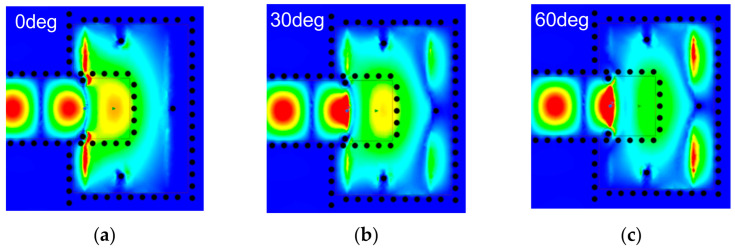
*E*-field distribution in the proposed transition with different phases: (**a**) Phase = 0°; (**b**) Phase = 30°; and (**c**) Phase = 60°.

The three-pole filter model presented in Figure 3 is employed to explain and guide the transition design. The desired transmission response can be obtained by an appropriate coupling matrix using the classical filter synthesis method [14]. The center frequency and bandwidth are set as 88.5 GHz and 25 GHz, respectively. The coupling coefficients are specified as follows: M*_S_*_2_ = 1.3374, M_11_ = −0.3313, M_12_ = 0.3934, M_13_ = 1.2432, M_22_ = −0.1532, M_23_ = 0.1953, M_33_ = 0.4023, M*_L_*_2_ = −1.0939, and M*_L_*_3_ = 0.7694. Additionally, two transmission zeros are caused by the cross-couplings of M*_L_*_2_ and M_23_. The S-parameter results of the equivalent model and full-wave simulation are demonstrated in Figure 4. The key parameters mentioned above are optimized to achieve the expected transition response. Table 1 gives the detailed dimensions of the optimized transition. As shown in Figure 4, the full-wave simulation result exhibits wideband performance, and it is in good agreement with the synthesis result of the equivalent three-pole filter model. The return loss of a single transition is better than 20 dB from 76 GHz to 101 GHz (28.2% bandwidth). Moreover, the *E*-field distribution with a different phase is shown in Figure 5. It is obvious that Resonator 2 and Resonator 4 are first excited by the input LWG, and they are coupled with Resonator 1. Then, the energy flows pass through Resonator 1 and couple to Resonator 3 and Resonator 5. This coupling path is the mainline coupling, and there are still other cross couplings in the transition. It can be demonstrated that the equivalent three-pole filter model is effective for the proposed transition scheme.

## 3. Fabrication and Measurement

**Figure 6:**
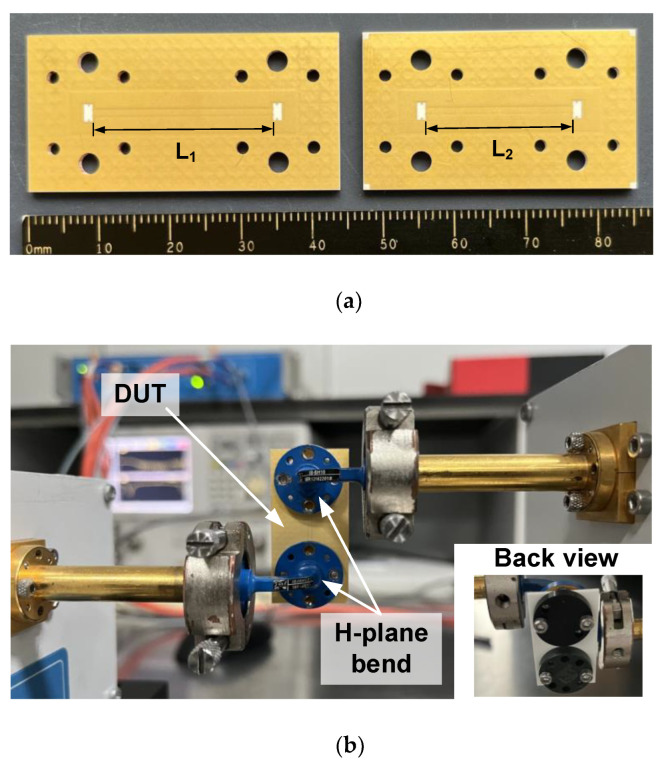
(**a**) Photograph of the fabricated back-to-back transitions. (**b**) Measurement setup.

To characterize the transmission performance of the single transition with different interfaces, two back-to-back prototypes with different lengths are fabricated and measured. Figure 6a is a photograph of two fabricated LTCC modules, in which the LWG lengths are, respectively, set to *L*_1_ = 25 mm and *L*_2_ = 20 mm. The back-to-back module is tested using a network analyzer combined with two W-band frequency extenders, and it is assembled with two H-plane 90° waveguide bends to facilitate measurement, as shown in Figure 6b. Four screws are used to tightly fix the LTCC module to the waveguide bends. The washers help to distribute the pressure to avoid cracking the LTCC substrate during measurement.

**Figure 7:**
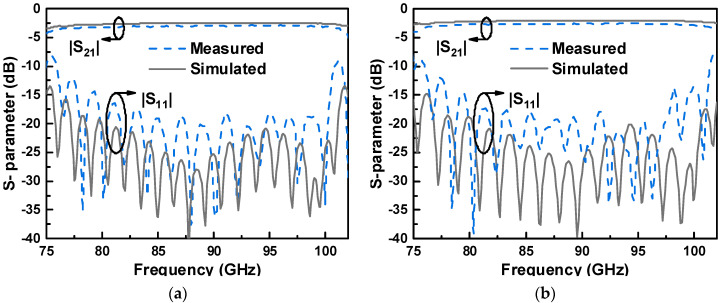
Simulated and measured results of two back-to-back transitions with an (**a**) LWG length of *L*_1_ and (**b**) an LWG length of *L*_2_.

The simulated and measured results of two back-to-back transitions are given in Figure 7. From the different measured insertion loss of two transitions, the transmission loss of the LTCC laminated waveguide can be estimated. The insertion loss of the back-to-back transition can be calibrated by subtracting the transmission loss of the LWG line, half of which is the insertion loss of the single transition. The measured results demonstrate that the return loss is better than 14 dB in the range from 78 GHz to 101 GHz (25.7% bandwidth). The measured insertion loss of two back-to-back transitions with different length are, separately, 3 dB and 2.6 dB on average, and the transmission loss of the 10 mm LWG is calculated as about 0.8 dB. Therefore, it can be derived that the calibrated insertion loss of a single transition is about 0.5 dB in the operating band. The slight discrepancy between the simulation and the measured results may be due to the fabrication tolerance, assembly errors, and leakage wave caused by the waveguide gap. Compared with the previous LTCC works listed in Table 2, the proposed transition in this paper exhibits great bandwidth and low-loss performance without compromising structure size.

## 4. Conclusions

A novel compact LWG-to-RWG transition with large bandwidth is presented for W-band LTCC integrations. Five grounded via holes are introduced to separate the transition cavity into several resonators. The working principle is interpreted by an equivalent three-pole filter model that is demonstrated to be effective for the proposed transition. The back-to-back prototypes are fabricated and measured with good performance. The compact size and wide bandwidth make the proposed transition applicable in LTCC integrated systems.

## Figures and Tables

**Table 1:** Dimension parameters of the proposed transition (unit: mm).

*L_C_*	*W_C_*	*L_S_*	*W_S_*	*D_V_*	*d_1_*	*d_2_*	*d_3_*
1.75	2.54	0.86	0.92	0.18	0.85	0.25	0.23

**Table 2:** Comparison with previous LTCC LWG-to-RWG transitions.

Reference	Frequency (GHz)	Insertion Loss (dB)	Return Loss (dB)	Fractional Bandwidth	Footprint (*λ_c_*^2^)
[10]	27.8–30.3	0.4	15	8.6%	0.73 × 0.69
[11]	34.8–37.8	0.26	13	8.3%	0.53 × 1.03
[12]	76–81	1.05	10	6.4%	0.29 × 0.41
This work	78–101	0.5	14	25.7%	0.52 × 0.75

## Data Availability

Not applicable.
